# Supplementary material for: A Global Screen for Assembly State Changes of the Mitotic Proteome by SEC-SWATH-MS
Source: Cell Syst. 2020 Feb 26;10(2):133–155.e6. doi: 10.1016/j.cels.2020.01.001 (PMC7042714; doi:10.1016/j.cels.2020.01.001)

P21399 | ACOC_HUMAN | ACO1 IREB1
Monomer MW [kDa]: 98.399 Monomer expected elution fraction: 40

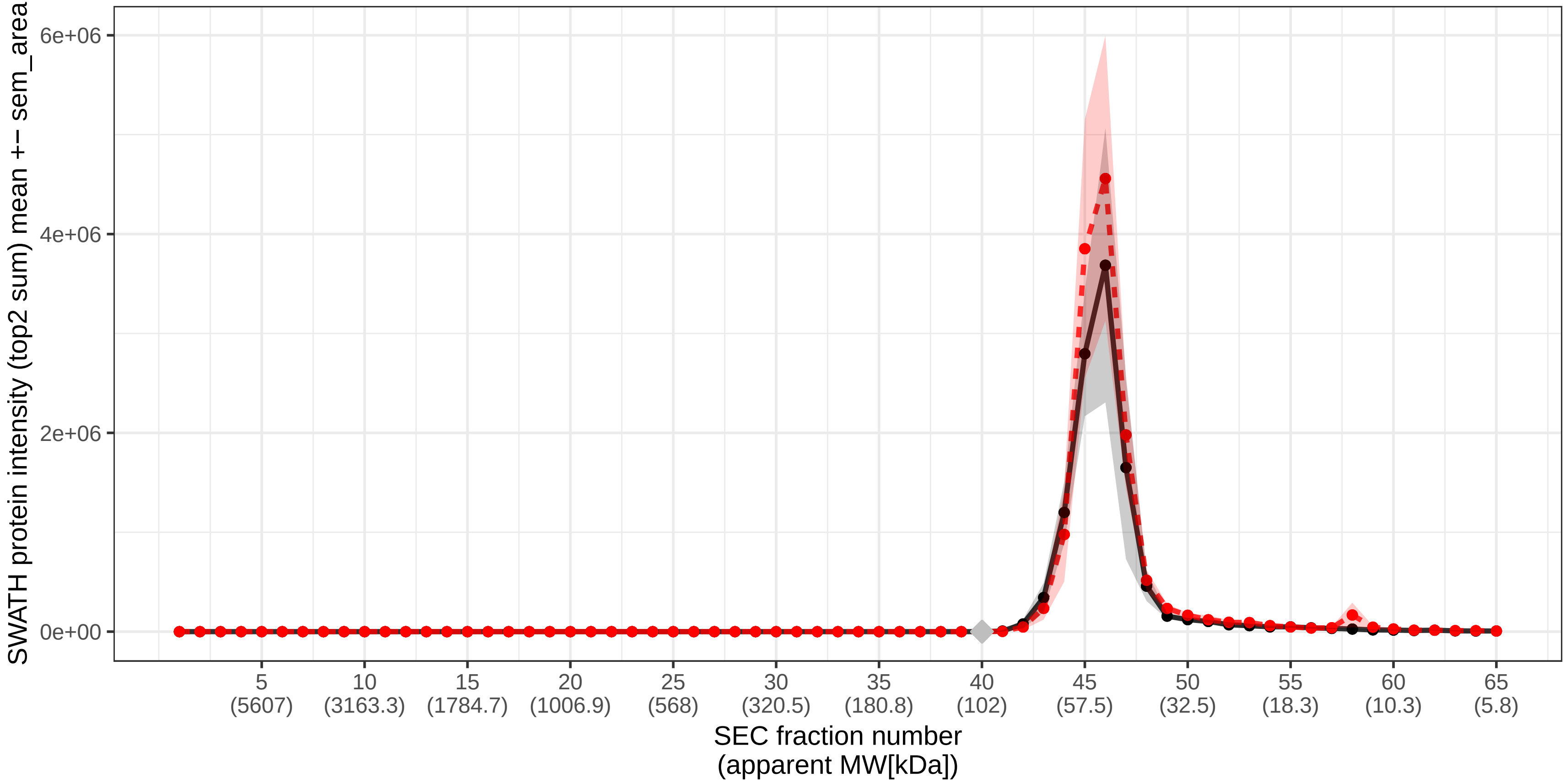

Supplement: Data S1. SEC-SWATH-MS Protein Chromatograms, Related to Figure 1 [file mmc6.zip › SECchrom_P21399_ACOC_HUMAN_ACO1_IREB1.pdf]
